# Newborn chicks need no number tricks. Commentary: Number-space mapping in the newborn chick resembles humans' mental number line

**DOI:** 10.3389/fnhum.2015.00451

**Published:** 2015-08-11

**Authors:** Samuel Shaki, Martin H. Fischer

**Affiliations:** ^1^Department of Behavioral Sciences, Ariel UniversityAriel, Israel; ^2^Division of Cognitive Sciences, University of PotsdamPotsdam, Germany

**Keywords:** mental number line, innate number sense, numerical cognition, spatial cognition, spatial numerical associations

The study of numerical ordering abilities in chicks recently reported by Rugani et al. (2015) concluded that “similarly to humans, chicks associate smaller numbers with the left space and larger numbers with the right space.” (p. 534). But do their results really show that 3-day-old domestic chicks are aware of both relative and absolute quantities, map them from left to right, and have an “innate number sense” (Rugani et al., 2015, p. 536)?

Rugani et al. (2015) trained newborn chicks to expect food behind a centrally presented occluder and tested them by presenting two laterally displaced occluders (see their Figure 1 below). The key manipulation was the amount of black dots shown on both training and test occluders. After seeing five dots at training (in Experiment 1), when the chicks saw two dots on both lateralized test occluders they walked to the left; while seeing eight dots on either side at test biased walking choices to the right. Similarly, after seeing 20 dots at training (in Experiment 2), seeing eight vs. 32 dots at test induced walking to the left vs. right side. Thanks to controlling for non-numerical factors such as area, perimeter and density of the dot patterns (Experiment 3), the authors concluded that newborn chicks associate relatively small numbers with left space and relatively larger numbers with right space, in analogy with a vast literature of spatial-numerical associations (SNAs) in humans (recently reviewed by Fischer and Shaki, 2014).

**Figure 1:**
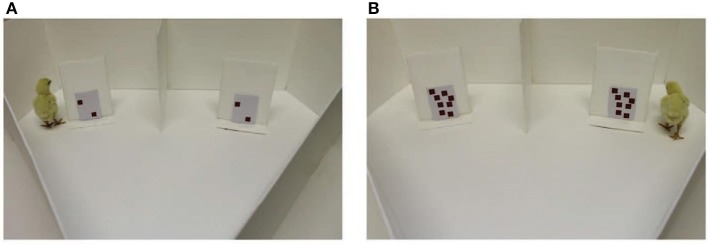
**(Figure re-used by permission from Rugani et al., 2015): The panels (A,B) differ in numerosity as well as lightness, density, perceptual mass, saliency, and visual complexity**.

This finding is deemed important because it challenges the view that language is the foundation of symbolic thought, in particular our ability to represent quantities beyond the span of immediate apprehension (subitizing range of 3–4 items; cf. Kaufman et al., 1949). Moreover, it questions the well-established cultural origin of horizontal SNAs (Shaki et al., 2009; Shaki and Fischer, 2014). Despite the endorsement of these points by Brugger (2015) in his associated perspective, we have reason to doubt these conclusions.

The authors acknowledged that the results of Experiments 1 and 2 could be accounted for by non-spatial cues as area, perimeter, or density of the displayed dots. They controlled for these confounds with numerosity in two conditions of their third experiment. Specifically, either perimeter and area were held constant, or perimeter and density were held constant, while numerosity varied. However, this means that numerosity co-varied either with density (in the first condition) or with area (in the second condition). Therefore, chicks in all control conditions responded to both numerosity and at least one uncontrolled dimension, as they were in the first and second experiments, too.

This methodological detail contains the core of our concern because both density and area are indeed spatially effective cues. Whenever object density was uncontrolled, fewer black dots resulted in reduced perceptual mass or saliency (see Figure 1). Both of these features are known to be associated with space (Christman and Pinger, 1997; Heath et al., 2005; Friedrich et al., 2014): Denser displays attract attention to the right and this may explain why chicks turn to the left in the presence of fewer dots compared to their training. Similarly, whenever object area was uncontrolled, fewer black dots resulted in lighter occluders (see Figure 1). There is an established association of light with left (Elias and Robinson, 2005; McDine et al., 2011); this “left-light bias” may explain why chicks turn to the left in the presence of fewer dots compared to their training. Together, these concerns clarify that “current ways to control the visual cues of the number stimuli are insufficient, as they control only a single variable at the [sic] time” (Gebuis and Reynvoet, 2012, p. 642).

A further point of concern is that less and more visually complex patterns are associated with left and right space, respectively (Beaumont, 1985; Heath et al., 2005). This feature co-varied with numerosity and was not even controlled here but it also can explain why chicks turned left or right when they encountered fewer or more dots, respectively, when compared to their training. Therefore, we require replications with reversed contrast and with complexity dissociated from numerosity (cf. Heath et al., 2005)—Will Rugani et al.'s result replicate in the latter and reverse in the former case? Both outcomes would falsify their conclusions.

To conclude, newborn chicks know no number tricks: several non-numerical spatial associations can parsimoniously account for the choices of chicks without invoking the concept of a spatially oriented mental number line. The considerable flexibility of SNAs in humans and their equivalent strength in either direction (Fischer and Shaki, 2014), the as well as some evidence from patients who neglect small numbers regardless of the lateralization of their spatial deficit (van Dijck et al., 2011, 2012; Aiello et al., 2012, 2013; Pia et al., 2012), argue further against the proposal of Rugani et al. and indicate that SNAs may instead reflect cultural conventions. SNAs obtain with centrally presented numerical information and even in the absence of lateralized responses (Gevers et al., 2010; Fischer and Shaki, 2015), thus making the mental number line a purely conceptual link between number and space.

## Conflict of interest statement

The authors declare that the research was conducted in the absence of any commercial or financial relationships that could be construed as a potential conflict of interest.
